# A prospective pilot study on plasma amyloid beta oligomers and postoperative delirium

**DOI:** 10.3389/fmed.2025.1673496

**Published:** 2025-10-27

**Authors:** YoungSoon Yang, Ki Jin Jung, Yong Tae Kwak

**Affiliations:** ^1^Department of Neurology, Cheonan Hospital, Soonchunhyang University College of Medicine, Cheonan, Republic of Korea; ^2^Department of Orthopedic Surgery, Cheonan Hospital, Soonchunhyang University College of Medicine, Cheonan, Republic of Korea; ^3^Department of Neurology, Hyoja Geriatric Hospital, Yongin, Republic of Korea

**Keywords:** Alzheimer’s disease, amyloid-beta oligomers, biomarkers, elderly patients, postoperative delirium

## Abstract

**Introduction:**

Postoperative delirium (POD) is common in older adults and has been linked to Alzheimer’s disease (AD). Plasma amyloid-β oligomers (AβOs) may clarify this relationship. We evaluated whether preoperative AβO burden is associated with POD severity.

**Methods:**

In this single-center prospective pilot study, we enrolled 22 patients aged ≥65 years undergoing hip or knee arthroplasty under general anesthesia. Blood was drawn preoperatively and postoperatively to quantify oligomerized amyloid-β using the multimer detection system (MDS-Oaβ). POD was assessed with the Korean version of the Delirium Rating Scale-98 (K-DRS-98). Group comparisons and correlations examined associations between MDS-Oaβ and POD.

**Results:**

Eleven of 22 patients developed POD. Those with POD were older and had higher preoperative MDS-Oaβ than those without POD (0.81 vs 0.56 ng/mL). There was no significant perioperative change in MDS-Oaβ, suggesting surgery or anesthesia did not alter the plasma Aβ oligomerization tendency. Within the POD group, preoperative MDS-Oaβ correlated with both K-DRS-98 severity and total scores.

**Discussion:**

In this pilot cohort, higher preoperative AβO burden was associated with the occurrence and severity of POD, while perioperative factors did not measurably affect AβO levels. These findings support a potential mechanistic link between AD-related pathology and POD. Given the small sample (*N*=22), estimates are imprecise and hypothesis-generating; validation in larger, multicenter studies is required before clinical application.

## 1 Introduction

Postoperative delirium (POD) is a prevalent and severe complication in elderly patients following surgical procedures under general anesthesia (1, 2). It is marked by acute disturbances in attention and cognition, often leading to extended hospital stays, increased morbidity and mortality, and long-term cognitive decline (3–6). Despite extensive research, the precise mechanisms of POD remain unclear. A prominent hypothesis suggests a link between POD and Alzheimer’s disease (AD). Preclinical and epidemiological evidence indicates a strong association between POD and AD (7). Inhalational anesthetics, such as isoflurane, have been implicated in the formation and aggregation of amyloid-beta (Aβ) protein and amyloid-beta oligomerization (8). Additionally, delirium is an independent risk factor for dementia, and patients with dementia are more likely to develop POD (9). Studies examining the relationship between POD and AD have primarily relied on cerebrospinal fluid (CSF) biomarkers or amyloid PET scans. However, these methods are costly and challenging to implement, limiting the ability to conduct large, diverse studies. Pilot studies using amyloid PET scans have reported inconsistent findings regarding brain amyloidosis and POD (10, 11). Meta-analyses show a negative correlation between CSF Aβ42 levels and POD occurrence, but no significant association with other CSF biomarkers (12). Recently, Payne et al. (13) published a study addressing this issue by utilizing plasma biomarkers, Aβ42 and Aβ40, which reflect cerebral amyloidosis. However, they were unable to find a significant association between these biomarkers and postoperative delirium. Recent reviews emphasize that postoperative delirium (POD) arises from multifactorial pathways and that perioperative biomarker signals are heterogeneous across studies and assay targets (14).

Within this landscape, blood measures targeting Aβ oligomerisation have shown diagnostic and pathophysiological relevance in Alzheimer’s disease and related amyloid pathology, supporting investigation of oligomer-focused assays in perioperative settings (15, 16). In contrast to these static measures of amyloid burden, emerging research suggests that oligomeric forms of Aβ (AβOs) may more accurately reflect ongoing pathological processes. Traditional biomarkers, such as Aβ42 or Aβ40 in the CSF, as well as amyloid PET imaging, capture relatively stable accumulations of amyloid over time. Aβ oligomers, however, are dynamic intermediates thought to appear earlier in disease progression, potentially signaling active synaptic dysfunction and neurotoxicity long before substantial plaque formation occurs (17). This enhanced sensitivity to active pathological changes may make oligomer-based assays more useful as predictive markers of conditions like POD, especially given their potential to capture subtle preclinical neurodegenerative changes.

In our recent retrospective study of 104 patients aged 70 and above undergoing general anesthesia, we measured blood levels of oligomerized amyloid-beta (MDS-Oaβ) using the multimer detection system (MDS) (18). The findings revealed notably elevated MDS-Oaβ levels in individuals who developed POD relative to those who did not. Furthermore, there was a strong positive association between MDS-Oaβ values and delirium severity, which persisted even after adjusting for the presence of the ApoE4 allele. These findings suggest a clear association between amyloid pathology and POD, distinguishing this study from previous ones that used different biomarkers. However, since that study was not a prospective study, there were limitations in confirming the causal relationship between MDS-Oaβ levels and POD.

To further investigate this association and explore the predictive potential of MDS-Oaβ values, we conducted a prospective pilot study. We aimed to determine how preoperative MDS-Oaβ values are related to POD.

## 2 Materials and methods

### 2.1 Study design and participants

This prospective, single-center observational pilot study was conducted at Soonchunhyang University Cheonan Hospital between July and December 2024. To reduce exposure heterogeneity and standardize peri-operative blood sampling, we enrolled 22 consecutive patients aged ≥65 years undergoing elective hip or knee arthroplasty under general anesthesia at a single center, reflecting predominant institutional practice in this elderly cohort and enabling consistent monitoring and sampling. Patients who were expected to be able to complete delirium testing postoperatively were selected for this investigation. Their global cognitive function was assessed using the Korean Mini-Mental State Examination (K-MMSE) on the day before surgery. The exclusion criteria were as follows: (1) patients with the MMSE score <24 or dementia due to various etiologies; (2) patients with daily tranquilizer or antipsychotic medication use and extensive alcohol consumption; (3) patients with known CNS diseases, such as psychiatric illnesses; (4) patients who could not complete the delirium testing, such as those who were expected to remain intubated postoperatively, particularly if they would be sedated for postoperative ventilation; (5) patients with hearing or visual deficits that would prevent neuropsychological testing. All study participants underwent routine blood tests and ApoE gene analysis. Given the pilot study nature and small sample size, we have presented detailed individual patient characteristics in Supplementary Table 1 to ensure transparency. This was an exploratory pilot; no *a priori* power calculation was performed. The sample size (*n* = 22) reflected feasibility within a fixed enrolment window and was intended to yield preliminary variance and effect-size estimates to inform sample-size planning for a larger multicenter study. This study received ethics committee approval and written informed consent from all participants (IRB #2023–02-038, Soonchunhyang University College of Medicine, Cheonan Hospital) and was conducted from February 2023 to April 2024. Written informed consent was obtained from each patient. All methods were performed in accordance with the relevant guidelines and regulations.

### 2.2 Detection of delirium and sample collection

Following surgery, participants were assessed for delirium daily for the first 3 days by trained research staff. If a patient in the ICU showed suspected delirium, the Korean Version of Delirium Rating Scale-98 (K-DRS-98) was obtained. Delirium diagnosis was based on the K-DRS-98 scale, where a severity score equal to or greater than 18.5, or a total score above 20.5, was used to indicate POD, according to previously published criteria (19). To ensure accuracy, daily consultations with nursing staff were conducted to corroborate findings. The patient underwent blood sampling twice: the first was done the day before surgery along with other routine blood tests, and the second was performed when POD occurred. If delirium did not occur within 3 days after surgery, the second sampling was done on the morning of the fourth postoperative day.

### 2.3 The measurement of oligomerization of Aβ in plasma

Oligomerization of Aβ in plasma was assessed using the MDS-Oaβ method (20, 21). This test was performed when delirium was suspected after surgery, while patients who did not show delirium undergo the tests fourth days postoperatively. Prior to the assay, plasma samples were initially thawed at a temperature of 37 °C for a duration of 15 min. After this, synthetic amyloid-beta (Aβ made by PeopleBio Inc.) was added to the samples, followed by an incubation period of 48 h at 37 °C. The incubated plasma sample mixture and serially diluted standard samples were added to their respective wells, and the plates were incubated at room temperature for 1 h. Subsequently, 100 μL/well of an enhanced chemiluminescence substrate solution (Rockland Immunochemicals Inc., Limerick, PA, USA) was added, and the Relative Luminescence Unit (RLU) values were determined using a Victor 3 spectrophotometer to quantify oligomerized Aβ.

### 2.4 Statistical analysis

Descriptive statistics were presented as median with interquartile range (IQR) for continuous variables and as number (percentage) for categorical variables. Analyses were descriptive; two-sided tests (*α* = 0.05) were used. Differences between patients with and without POD were assessed using chi-square tests for categorical variables (and Mann–Whitney U test for continuous variables and Pearson’s chi-square or, where expected cell counts were <5, two-sided Fisher’s exact tests for categorical variables). For key contrasts, we report effect estimates with 95% confidence intervals to convey precision; no adjusted or causal models were specified. Finally, we used Spearman’s rank correlation coefficient analysis to determine the relationship between MDS-Oaβ values and the severity of delirium, as defined by the K-DRS-98 scores. Statistical analyses were performed using SPSS version 24.0 (SPSS, Inc., Chicago, IL, USA).

## 3 Results

### 3.1 Clinical characteristics and the variables relating delirium in all study participants, patients with and without postoperative delirium

After applying inclusion and exclusion criteria, twenty-two patients were included as index cases for analysis (Table 1). Table 1 summarizes the baseline clinical characteristics and MDS-Oaβ levels of all study participants, with a specific focus on comparing those who developed POD and those who did not. The median of the overall study population was 75.50 (71.25–78.75) years, with a higher median age observed in the POD group [79.00 year (74.00–80.50)] compared to the non-POD group [74.00 years (69.00–76.00)] (*p* = 0.025). Female participants constituted 36.4% of the total population, with same proportions in both the POD and non-POD groups. Cognitive function, as measured by the Mini-Mental State Examination (MMSE), was significantly lower in the POD group [28.00 (26.50–28.00)] compared to the non-POD group [29.00 (28.50–29.00)]. There was no statistically significant difference in the number of ApoE4 alleles between the POD and non-POD groups. Preoperative MDS-Oaβ levels were significantly elevated in the POD group [0.81 ng/mL (0.69–0.89)] compared to the non-POD group [0.56 ng/mL (0.35–0.68)]. Postoperative levels of MDS-Oaβ showed no significant difference from preoperative values.

**Table 1:** Clinical characteristics and MDS-Oaβ value in the cverall study population, patients with and without postoperative delirium.

Variables	Overall (*N* = 22)	Delirium		*p*-value*
		Yes, *N* = 11	No, *N* = 11	
Age, years	75.50 (71.25–78.75)	79.00 (74.00–80.50)	74.00 (69.00–76.00)	0.025
Female gender (%)	8 (36.4%)	4 (36.4%)	4 (36.4%)	1.0
Education	9.00 (6.50–10.00)	9.00 (6.00–10.00)	9.00 (8.50–12.00)	0.125
MMSE	28.00 (27.25–29.00)	28.00 (26.50–28.00)	29.00 (28.50–29.00)	0.003
Number of ApoE4 gene	0.00 (0.00–0.00)	0.00 (0.00–0.50)	0.00 (0.00–0.00)	0.559
Surgery name				
THRA	14	7	7	
CR and IF of femur	3	2	1	
ARCR	2	1	1	
Others	3	1	2	
K-DRS-98 severity	13.00 (4.25–23.00)	23.00 (21.00–26.50)	4.00 (3.00–6.00)	0.000
K-DRS-98 total	15.00 (4.25–25.75)	26.00 (24.50–30.00)	4.00 (3.00–6.00)	0.000
preMDS-Oaβ (ng/mL)	0.69 (0.44–0.80)	0.81 (0.69–0.89)	0.56 (0.35–0.68)	0.013
postMDS-Oaβ (ng/mL)	0.69 (0.45–0.82)	0.84 (0.70–0.89)	0.56 (0.38–0.69)	0.011
delta MDS-Oaβ (ng/mL)	0.01 (0.00–0.02)	0.01 (0.01–0.02)	0.00 (0.00–0.01)	0.180

Values are median (IQR) for continuous variables and *n* (%) for categorical variables.

THRA, Total Hip Replacement Arthroplasty; CR and IF of femur, closed reduction and internal fixation of femur, ARCR, Arthroscopic Rotator Cuff Repair; K-DRS-98, Korean Version of Delirium Rating Scale-98; MDS-Oaβ, Multimer Detection System-Oligomeric Amyloid-β. ^*^Statistical test was done between patients with POD and without POD.

### 3.2 The relationship between the MDS-Oaβ values and severity of delirium in study subjects

In study subjects, preoperative MDS-Oaβ and postoperative values showed a positive correlation with both K-DRS-98 severity (*p* < 0.01) and K-DRS-98 total scores (*p* < 0.01) (Table 2).

**Table 2:** Spearman correlation coefficient matrix among K-DRS and preMDS-Oaβ, postMDS in study subjects (*n*=22).

Variables	preMDS-Oaβ (ng/mL)	postMDS-Oaβ (ng/mL)	K-DRS-98 severity	K-DRS-98 total
preMDS-Oaβ (ng/mL)	1.0	0.984^**^	0.625^**^	0.605^**^
postMDS-Oaβ (ng/mL)		1.0	0.634^**^	0.616^**^
K-DRS-98 severity			1.0	0.993^**^
K-DRS-98 total				1.0

*Statistical significance p-value was lower 0.05. ** Statistical significance *p*-value was lower 0.01.

## 4 Discussion

As the population ages, the number of surgeries requiring general anesthesia is increasing, and with it, the incidence of delirium associated with these procedures is also rising rapidly. Numerous studies have revealed how frequently this condition occurs and the significant issues associated with it. However, the exact cause of POD remains elusive, complicating efforts to predict, prevent, and treat it (4, 9). Among the many hypotheses, the most compelling one is that POD is associated with AD (11, 22). Clinically, the patient may appear normal without signs of dementia, but there exists AD-related pathology in the brain (preclinical AD). In this condition, the brain may become highly vulnerable to factors that can negatively affect it, such as surgery, which may trigger POD (9).

Our prospective pilot study aimed to address this hypothesis by using the MDS-Oaβ value, a biomarker reflecting Aβ oligomerization (AβOs), which are more neurotoxic and dynamic than static amyloid plaques. Our previous retrospective study showed that MDS-Oaβ values measured when patients developed postoperative delirium were significantly higher compared to those without delirium, and these values were positively correlated with the severity of delirium. These results prompted an urgent need for a prospective pilot study to determine whether MDS-Oaβ values are elevated before surgery, how surgery affects these values, and ultimately whether this test can serve as a biomarker to predict POD preoperatively.

Our study demonstrated that preoperative MDS-Oaβ levels were markedly elevated in patients who developed POD, and there was no significant change in MDS-Oaβ values before and after surgery, suggesting that general anesthesia or surgery itself does not affect these levels. These results support the hypothesis that preoperative MDS-Oaβ values are linked to POD and may indicate underlying brain amyloidosis or preclinical AD (23). Additionally, the results of this study showed that patients who developed POD were statistically older and had lower MMSE scores than those without POD, suggesting that the patients with POD may have been affected by AD-like pathology. Although it would have been necessary to adjust for MMSE scores and age as potential confounding factors when comparing MDS-Oaβ values, the small sample size inherent in this pilot study limits the statistical power of such adjustments. For simplicity and clarity, we have not included these analyses in the main text, but they are provided in Supplementary Table 2 and refrain from asserting independent prediction by MDS-Oaβ, treating those estimates as exploratory.

However, since the MDS-Oaβ values did not show significant differences before and after the surgery, it is unlikely that changes in plasma amyloid beta oligomerization, or newly developed amyloidosis related to the surgery, caused the POD. The preoperative MDS-Oaβ values suggest underlying vulnerability in the brain before surgery. While increased Aβ oligomerization might lower the threshold for delirium, other factors—such as heightened inflammatory responses induced by surgical trauma, imbalances in key neurotransmitters (e.g., cholinergic and dopaminergic systems), and the specific type and depth of anesthesia employed—also interact to shape the risk and severity of POD. Aβ oligomers might set the stage, but the final manifestation of delirium likely emerges from the combined impact of these diverse physiological and environmental influences.

**Figure 1:**
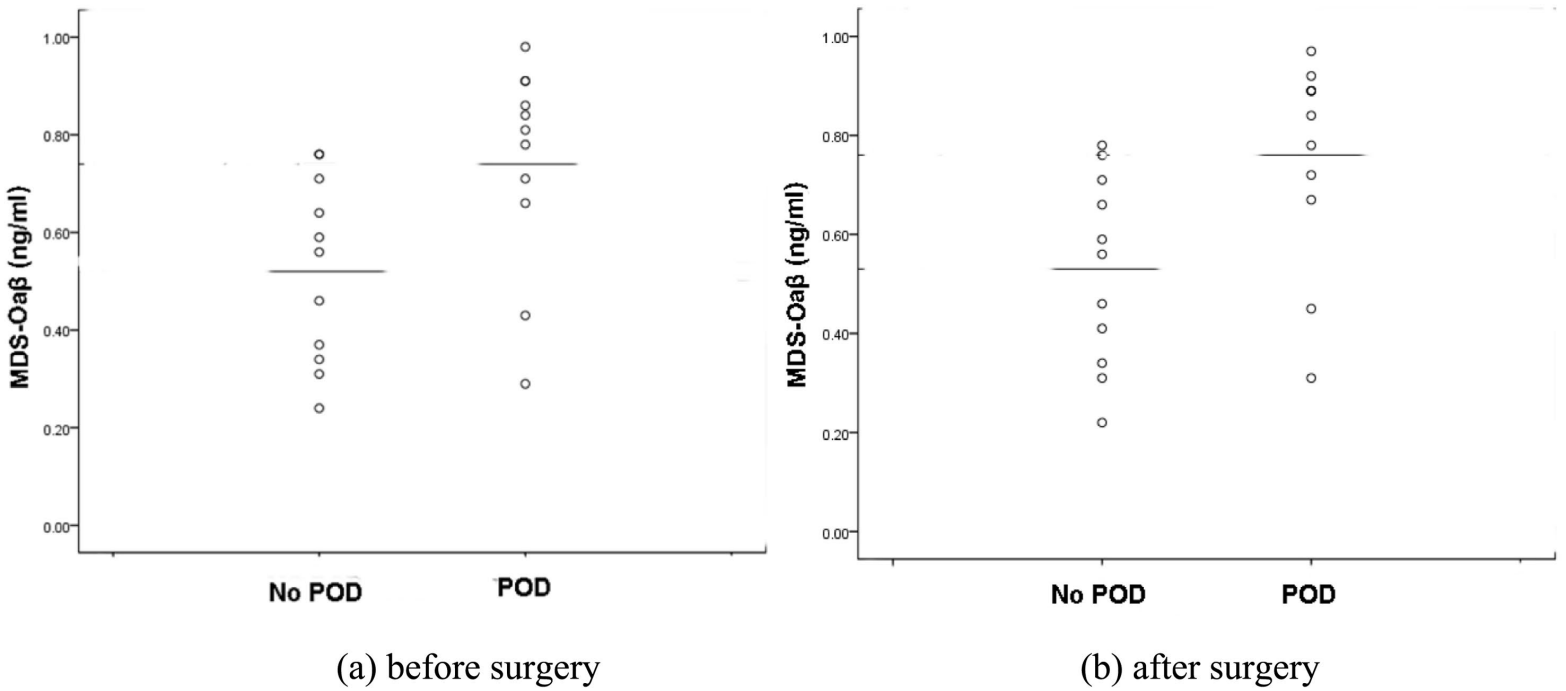
Scatter plots of plasma MDS-Oaβ (ng/mL) levels in patients with and without POD. **(a)** Plasma MDS-Oaβ levels sampled before surgery. **(b)** Plasma MDS-Oaβ levels sampled after surgery. In both scatter plots, the median MDS-Oaβ levels between POD and No POD groups are statistically significant (*p* < 0.05). POD; postoperative delirium.

In this study, as shown in Figure 1, although the preoperative and postoperative levels of MDS-Oaβ values were statistically significantly higher in the patients with POD, there were two cases among the patients who experienced POD where the MDS-Oaβ values were exceptionally low, unlike the other patients. To identify the characteristics of these patients, the authors reviewed all the study data and tracked the two patients. The first case is a 72-year-old male patient with a preoperative MDS-Oaβ level of 0.29 who developed POD. This patient had a history of lung cancer and underwent internal fixation for a femur fracture. Despite having no neurological or dementia symptoms before surgery, the patient developed severe delirium postoperatively and a Brain MRI performed 7 days after surgery revealed lung cancer brain metastasis. The second case is a 78-year-old female patient with a preoperative MDS-Oaβ level of 0.43. She also had no prior history of dementia or neurological symptoms and underwent internal fixation surgery for a femur fracture. Postoperatively, the patient developed pneumonia, which required ongoing treatment. In the first case, the patient was included in the study because the pre-existing brain condition was not known beforehand. In the second case, pneumonia developed right after the evaluation was completed following the occurrence of POD. Since there were no pneumonia symptoms at the time of the evaluation, the patient was included in the study. However, it is possible that pneumonia, despite being asymptomatic during the evaluation, might have already had some impact on the brain. These two cases illustrate that delirium can arise from diverse causes. While elevated amyloid oligomers may increase the risk of POD, other factors can also contribute. This reminds us that POD is a complex condition requiring a multifaceted approach to both study and treatment. Although these two cases were included in the study, the MDS-Oaβ values were still significantly higher in the patients with POD. If these two cases were excluded from the analysis, the difference between the two groups would be more pronounced, making the causal relationship between this value and POD clearer. However, this point is offered only for context. The cases are anecdotal and do not warrant causal inference about MDS-Oaβ and POD.

Although this study has addressed many of the gaps from previous research, there are still several limitations. First, this was a single-center, prospective pilot study with a relatively small sample size (22 participants), which may limit the statistical power of our findings. As a result, the observed associations between preoperative MDS-Oaβ values and postoperative delirium (POD) must be interpreted cautiously. Additionally, we could not present MDS-Oaβ values with meaningful sensitivity and specificity for predicting POD. Second, because the study was conducted at a single institution and involved a specific surgical population (primarily patients undergoing hip or knee arthroplasty), the external validity of our results may be restricted. The applicability of these findings to other types of surgery, different patient populations, or varying healthcare settings is not yet established. Accordingly, findings are preliminary and hypothesis-generating; a multicenter study with *a priori* sample-size justification is planned.

To overcome these limitations, we plan to expand this research by conducting a large-scale, multi-center prospective study involving various surgical patients. The goal is to obtain reliable results on the relationship between preoperative MDS-Oaβ values and postoperative delirium. Furthermore, we aim to propose MDS-Oaβ values as a reliable biomarker for predicting postoperative delirium. This would enable the comprehensive assessment of not only the surgery itself but also the associated risk of delirium in elderly patients requiring general anesthesia, thereby aiding in the overall decision-making process for surgery.

## Data Availability

The original contributions presented in the study are included in the article/Supplementary material, further inquiries can be directed to the corresponding author.
