# Supplementary material for: A prospective pilot study on plasma amyloid beta oligomers and postoperative delirium
Source: Front Med (Lausanne). 2025 Oct 27;12:1673496. doi: 10.3389/fmed.2025.1673496 (PMC12612744; doi:10.3389/fmed.2025.1673496)
Supplement: Supplementary file 1 [file Table_1.docx]

**Supplementary Table 1. Clinical characteristics of study subjects**

| ID | Sex | Age | ApoE | Op name | preMDS-Oaβ | postMDS-Oaβ | Delirium | K-DRS-98 severity | K-DRS-98 total |
| --- | --- | --- | --- | --- | --- | --- | --- | --- | --- |
| 1 | M | 69 | 3/3 | THRA Lt. | 0.34 | 0.41 | No | 3 | 3 |
| 2 | M | 72 | 3/4 | THRA Rt. | 0.91 | 0.92 | Yes | 24 | 28 |
| 3 | F | 78 | 3/3 | TKRA | 0.56 | 0.56 | No | 5 | 5 |
| 4 | F | 79 | 3/3 | arthroscopic meniscal repair | 0.84 | 0.89 | Yes | 29 | 33 |
| 5 | M | 69 | 3/3 | ARCR | 0.59 | 0.59 | No | 2 | 2 |
| 6 | M | 81 | 4/4 | THRA Lt. | 0.98 | 0.97 | Yes | 28 | 33 |
| 7 | M | 78 | 3/3 | THRA Rt. | 0.64 | 0.66 | No | 3 | 4 |
| 8 | F | 82 | 3/3 | THRA Lt. | 0.66 | 0.67 | Yes | 23 | 25 |
| 9 | F | 75 | 2/3 | THRA Rt. | 0.71 | 0.71 | No | 6 | 6 |
| 10 | M | 76 | 3/3 | THRA Rt. | 0.81 | 0.84 | Yes | 19 | 23 |
| 11 | M | 68 | 3/3 | THRA Lt. | 0.46 | 0.46 | No | 2 | 2 |
| 12 | M | 80 | 3/3 | THRA Rt. | 0.78 | 0.78 | Yes | 20 | 23 |
| 13 | M | 75 | 3/4 | CR and IF of femur | 0.76 | 0.76 | No | 7 | 7 |
| 14 | F | 84 | 3/4 | THRA Rt. | 0.71 | 0.72 | Yes | 26 | 30 |
| 15 | F | 77 | 3/3 | THRA Lt. | 0.31 | 0.31 | No | 4 | 4 |
| 16 | M | 72 | 3/3 | CR and IF of femur | 0.43 | 0.45 | Yes | 20 | 25 |
| 17 | F | 69 | 3/3 | Excision and biopsy Mass, thigh Lt. | 0.37 | 0.34 | No | 3 | 3 |
| 18 | M | 74 | 3/4 | THRA Lt. | 0.24 | 0.22 | No | 6 | 6 |
| 19 | F | 78 | 3/3 | CR and IF of femur | 0.29 | 0.31 | Yes | 23 | 26 |
| 20 | M | 71 | 3/3 | THRA Lt. | 0.76 | 0.78 | No | 6 | 6 |
| 21 | M | 68 | 3/3 | ARCR | 0.86 | 0.89 | Yes | 22 | 24 |
| 22 | M | 79 | 3/3 | THRA Lt. | 0.91 | 0.89 | Yes | 27 | 30 |
|  |  |  |  |  |  |  |  |  |  |

THRA: Total Hip Replacement Arthroplasty, TKRA; Total Knee Replacement Arthroplasty, CR and IF of femur ; closed reduction and internal fixation of femur, ARCR; Arthroscopic Rotator Cuff Repair, K-DRS-98; Korean Version of Delirium Rating Scale-98, MDS-Oaβ: Multimer Detection System-Oligomeric Amyloid-β
