# Supplementary material for: A prospective pilot study on plasma amyloid beta oligomers and postoperative delirium
Source: Front Med (Lausanne). 2025 Oct 27;12:1673496. doi: 10.3389/fmed.2025.1673496 (PMC12612744; doi:10.3389/fmed.2025.1673496)
Supplement: Supplementary file 2 [file Table_2.docx]

**Supplementary Table 2. Results of logistic regression analysis predicting POD(n=22).**

| Variables | B | SE(B) | Exp(B) | p-value |
| --- | --- | --- | --- | --- |
| Age | 0.462 | 0.251 | 1.587 | 0.066 |
| Education | -0.102 | 0.398 | 0.903 | 0.799 |
| MMSE | -2.294 | 0.992 | 0.101 | 0.021 |
| Number of ApoE4 gene | -1.714 | 1.961 | 0.180 | 0.382 |

During the initial analysis, we included sex, preMDS-Oaβ, and postMDS-Oaβ, but these variables contributed to multicollinearity and overfitting due to the small sample size (N=22). Further analysis showed these variables were not significant predictors, so we excluded them to improve model stability, resulting in more reliable estimates and a simpler model focused on key predictors like MMSE, Age, Education, and ApoENumber.
